# Membrane vesicle-mediated bacterial communication

**DOI:** 10.1038/ismej.2017.13

**Published:** 2017-03-10

**Authors:** Masanori Toyofuku, Kana Morinaga, Yohei Hashimoto, Jenny Uhl, Hiroko Shimamura, Hideki Inaba, Philippe Schmitt-Kopplin, Leo Eberl, Nobuhiko Nomura

**Affiliations:** 1 Department of Life and Environmental Sciences, University of Tsukuba, Tsukuba, Ibaraki, Japan; 2 Department of Plant and Microbial Biology, University of Zurich, Zürich, Switzerland; 3 Technology Research Center, Sumitomo Heavy Industries, Ltd., Yokosuka, Kanagawa, Japan; 4 Helmholtz Zentrum München-German Research Center for Environmental Health, Research Unit Analytical BioGeoChemistry, Neuherberg, Germany; 5 Development and Analysis Center, Sumitomo Heavy Industries Environment Co., Ltd., Yokosuka, Kanagawa, Japan; 6 Technische Universität München, Chair of Analytical Food Chemistry, Freising, Germany

## Abstract

The classical quorum-sensing (QS) model is based on the assumption that diffusible signaling molecules accumulate in the culture medium until they reach a critical concentration upon which expression of target genes is triggered. Here we demonstrate that the hydrophobic signal *N*-hexadecanoyl-L-homoserine lactone, which is produced by *Paracoccus* sp., is released from cells by the aid of membrane vesicles (MVs). Packed into MVs, the signal is not only solubilized in an aqueous environment but is also delivered with varying propensities to different bacteria. We propose a novel MV-based mechanism for binary trafficking of hydrophobic signal molecules, which may be particularly relevant for bacteria that live in open aqueous environments.

Many bacteria employ small secreted molecules to communicate with each other, a phenomenon often referred to as quorum sensing (QS). Among the various bacterial signals identified to date, *N*-acyl-homoserine lactones (AHLs) are the most common QS signals produced by Gram-negative bacteria. The production of AHLs with *N*-acyl side chains containing 4–18 carbons and various additional modifications have been described in >200 Gram-negative bacterial species (Wagner-Döbler *et al.*, 2005). The classic QS model is based on the assumption that AHLs diffuse from the cell to the medium and back into the cell, and that it is the population density (that is equivalent with a critical AHL concentration) that triggers the QS regulatory cascade. However, free diffusibility was only demonstrated for the short-chain AHL *N*-butyryl-L-homoserine lactone (C4-HSL) and there is increasing evidence that AHLs containing longer fatty acid chains require transporters to be released from the cell (Pearson *et al.*, 1999; Chan *et al.*, 2007; Buroni *et al.*, 2009). It has also been reported that long-chain AHLs, like *N*-hexadecanoyl-L-homoserine lactone (C16-HSL), which are typically employed by rhizobial and roseobacterial QS systems, partition with the cell envelope (Blosser-Middleton and Gray, 2001; Marketon *et al.*, 2002; Schaefer *et al.*, 2002; Wagner-Döbler *et al.*, 2005; Barth *et al.*, 2012). Recent research has highlighted the importance of membrane vesicles (MVs) for the transfer of various cellular components between cells. MV production has been demonstrated for many bacteria and recent work has also provided evidence that they are abundant in natural environments (Biller *et al.*, 2014). These MVs have important roles in microbial and host-microbial interactions, delivering proteins and DNA (Brown *et al.*, 2015; Kaparakis-Liaskos and Ferrero, 2015; Schwechheimer and Kuehn, 2015). The opportunistic pathogen *Pseudomonas aeruginosa* has been demonstrated to package the signaling molecule 2-heptyl-3-hydroxy-4-quinolone (*pseudomonas* quinolone signal; PQS) into membrane vesicles that serve to traffic this molecule within a population (Mashburn and Whiteley, 2005).

In this study, we demonstrate that the very hydrophobic C16-HSL signal, which is used by *Paracoccus denitrificans* Pd1222 for cell-to-cell communication (Schaefer *et al.*, 2002), is released from the cells mainly by the aid of MVs. Moreover, we demonstrate that the MVs fuse with varying propensities to different bacteria, suggesting that the MVs are capable of recognizing particular cell types.

Spherical structures that are typical for MVs were observed in the supernatant of stationary phase *Paracoccus denitrificans* Pd1222 cultures grown in tryptic soy broth medium at 37 °C with shaking (Figure 1a). Given that the C16-HSL produced by this organism is more hydrophobic than PQS (logP of 6.05 versus 3.60, where P is the octanol-water partition coefficient), we hypothesized that this AHL signal may be associated with MVs. Culture supernatants of Pd1222 were ultracentrifuged to separate MV-associated from free C16-HSL and the MV pellet was further fractionated by density gradient ultracentrifugation. Only fractions containing MVs activated the AHL biosensor (Someya *et al.*, 2009), indicating that C16-HSL is associated with MVs (Supplementary Figure S1). The AHL biosensor was not activated when MV samples of an AHL-null mutant, in which the *luxI* homolog Pden_0787 of Pd1222 had been inactivated, were tested (Figure 1b). The Pden_0787 mutant no longer produced AHLs and we named this gene *pdnI* (*Paracoccus denitrificans luxI*-homolog) (Supplementary Figure S2). These results indicate that the C16-HSL that is associated with these MVs is biologically active and can induce gene expression in a target cell.

Although the functions controlled by C16-HSL in *P. denitrificans* have not been identified, we observed that the *pdnI* mutant strongly aggregates when grown at 30 °C. C16-HSL or wild-type MVs inhibited cell aggregation, while MVs of the *pdnI* mutant did not (Figure 1c). To examine whether MV-transported C16-HSL is delivered into Pd1222 cells, a GFP-based AHL reporter plasmid was introduced into a Pd1222 *pdnI* mutant. Addition of MVs to the medium induced GFP expression of the strain, suggesting that MVs that fuse with bacterial cells release their C16-HSL cargo to the cells (Figure 1d). These results indicate that C16-HSL is transported *via* MVs to control self-aggregation of Pd1222. Both inhibition of cell aggregation and GFP induction of the Pd1222 AHL reporter required at least 5 nM of exogenously added C16-HSL and the effects were maximal at 100–500 nM. The amount of MVs that was required to elicit expression of both phenotypes corresponded to 50 nM C16-HSL and a maximal response was observed at a concentration equivalent to 500 nM C16-HSL (Supplementary Figure S3).

*Paracoccus* species isolated from activated sludge also showed MV-associated AHL production (Supplementary Figure S4). Chemical analysis of the supernatant of strain AS6, identified C16-HSL as the major AHL signal (Supplementary Figure S5). MV-like structures could also be observed in activated sludge samples (Supplementary Figure S6). However, we were not able to detect AHLs from these MVs, presumably because the majority of these MVs are derived from species other than *Paracoccus* sp.

Quantification of C16-HSL by ultrahigh performance liquid chromatography coupled to time of flight mass spectrometry (UHPLC-qToF-MS) (Buddrus-Schiemann *et al.*, 2014), revealed that a late-stationary phase culture of Pd1222 produced ~2.5 μM of 16-HSL, of which 64% was released to the growth medium while the remainder was associated with the cells (Figure 1e). Of the extracellular C16-HSL, 53% were found to be associated with MVs. Both MV-associated and free C16-HSL in the supernatant was able to inhibit aggregation and induce GFP expression in the Pd1222 AHL reporter strain. This indicates that in addition to free AHLs (as in the classic QS model), *P. denitrificans* can also use MV-associated signals to trigger the QS response and that both systems appear to operate in parallel (Supplementary Figure S7). It is worth noting that C16-HSL was hardly detectable by UHPLC-qToF-MS in MV samples unless they were extracted with ethyl acetate (Figure 1e), indicating that C16-HSL is tightly bound to MVs. The negligible amounts of C16-HSL observed in untreated MV preparations were likely extracted from MVs by acetonitrile during the UHPLC, rather than being a contamination of the MV preparation with free C16-HSL. In a late-stationary-phase culture, 5.4 × 10^9^ MV particles per ml were detected. Assuming an equal distribution of the signal molecule and similar sizes of the MVs, each MV is associated with ~1.1 × 10^5^ C16-HSL molecules. The threshold concentration of free C16-HSL required for biological activity was determined to be 5 nM, which corresponds to a cell density of 8.5 × 10^9^ ml^−1^ (Supplementary Figure S3). Assuming a cell volume of 1 μm^3^, 3–350 C16-HSL molecules per cell are sufficient to trigger the QS cascade. Hence, the amount of C16-HSL associated with one MV is very likely high enough to induce the QS response in a *P. denitrificans* cell when it fuses with a MV.

As C16-HSL was tightly associated with MVs, we hypothesized that if C16-HSL is released *via* MVs, stimulation of MV production would increase the amount of C16-HSL in the supernatant. To test this hypothesis, we first examined whether MV formation by *P. denitrificans* can be stimulated by treatment with the DNA damaging agent mitomycin C (MMC) (Turnbull *et al.*, 2016). The amount of MVs released into the supernatant was found to be directly proportional to the MMC concentration that is added to the culture (Figure 1f). Previous work has shown that stress-induced MV formation in *P. aeruginosa* is dependent on *recA* (Toyofuku *et al.*, 2014; Turnbull *et al.*, 2016). In accordance, we observed that *recA* is also required for stress-induced MV formation in *P. denitrificans* (Figure 1f). We also observed that the amount of C16-HSL in the supernatant linearly increased (*R*^2^=0.98) with the MMC concentration (Figure 1g). In contrast, MMC treatment did not affect C16-HSL concentration in the supernatant of the *recA* mutant (Figure 1g). Importantly, C16-HSL production was not altered by MMC treatment, indicating that C16-HSL release but not its production was stimulated (Supplementary Figure S8). These data indicate that the release of C16-HSL depends on the production of MVs.

When C16-HSL was added to PBS in a polypropylene tube, it adsorbed firmly to the surface of the tube. In contrast, MV-associated C16-HSL could be easily recovered from the tubes (Supplementary Figure S9), indicating that the hydrophobic C16-HSL can be solubilized by MVs in aqueous systems. We next examined whether MVs would traffic C16-HSL to non-self cells. As an alternative target we employed the well-studied MV producer *P. aeruginosa.* MVs of *P. denitrificans* showed a low affinity for *P. aeruginosa* relative to *P. denitrificans* (Figures 2a and b). Importantly, in the *P. aeruginosa* background, the AHL reporter plasmid responded poorly to MV-associated C16-HSL compared to the addition of an equivalent amount of free signal molecule to the medium (Figure 2c). *P. denitrificans* responded well to both free and MV-associated C16-HSL (Figure 2d). We also observed that several other bacteria responded only weakly to MV-associated C16-HSL relative to free C16-HSL (Supplementary Figure S10). These results suggest that the cargo carried by *P. denitrificans* MVs is delivered with varying propensities to other bacteria. Further work will be required to unravel the underlying mechanism that determines the specificity of MV-associated AHL delivery.

Although the QS paradigm assumes free diffusibility of the signal molecule, evidence has accumulated that long-chain AHLs (Schaefer *et al.*, 2002; Wagner-Döbler *et al.*, 2005; Chang *et al.*, 2012) are not diffusing out of the cell (Barth *et al.*, 2012; Krol and Becker, 2014), in contrast to short-chain AHLs (Pearson *et al.*, 1999). At present, very little is known how these hydrophobic signal molecules are released by the cell. A recent study of a marine *Vibrio* suggested that MVs can induce AHL-regulated gene expression, although the signal molecule has not been identified (Li *et al.*, 2016). Here we show that long-chain AHLs are associated with MVs, which not only allow for their secretion but also guide their transport to specific target cells. When packed into MVs, the hydrophobic C16-HSL can be solubilized in an aqueous environment. Our data suggest that long-chain AHLs are concentrated in MVs, which will ensure that the amount of signals delivered to a target cell is sufficient to trigger its QS response. This binary signaling mechanism is fundamentally different from the classic QS model, which assumes the analog accumulation and homogenous distribution of the signal in the medium until a critical concentration is reached that induces the QS response synchronously in the majority of cells. Our data show that in a closed test tube system the amount of free AHL is sufficiently high to trigger a classical QS response and although the MV-based signaling will operate in parallel in this system, it would not be essential for QS induction. We therefore propose that the MV-based signaling is particularly valuable for trafficking hydrophobic signal molecules in natural habitats, particularly for bacteria that live in open aqueous environments where non-MV associated signals would be infinitely diluted.

## Figures and Tables

**Figure 1:**
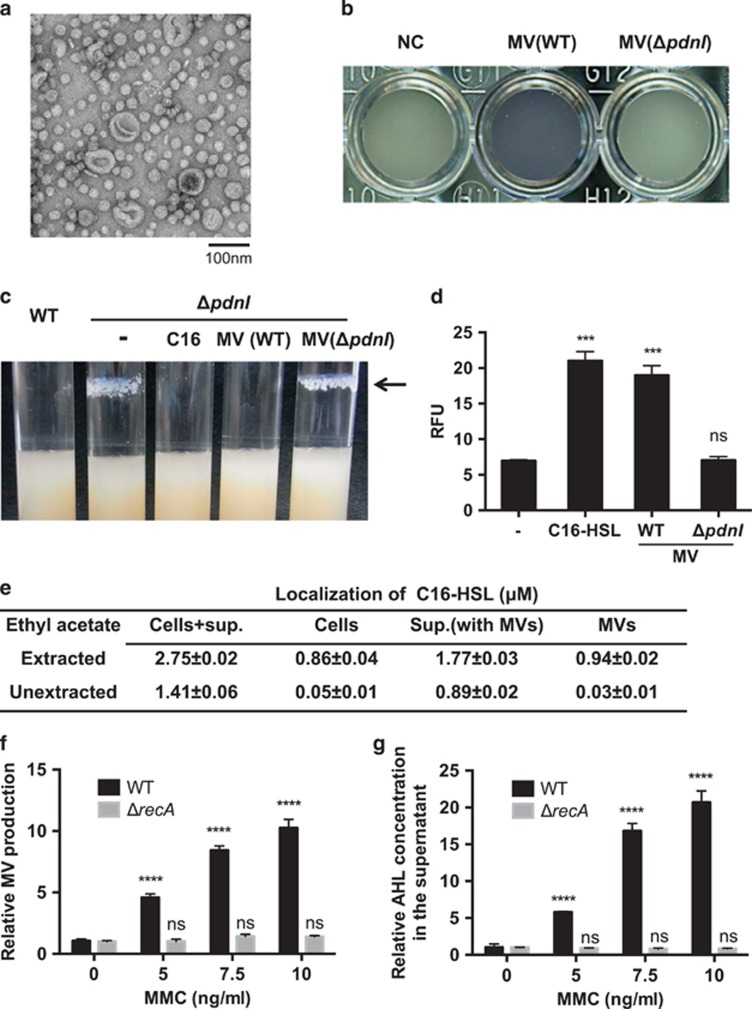
C16-HSL are associated with MVs in *P. denitrificans* Pd1222. (**a**) TEM image of MVs isolated from Pd1222. (**b**) *C. violaceum* VIR24 assay for the detection of C16-HSL. MVs isolated from Pd1222 wild-type or a *pdnI* mutant were analyzed. The purple pigment is indicative of the presence of C16-HSL. (**c**) C16-HSL inhibits Pd1222 aggregation. Arrows indicate cell aggregates of the *pdnI* mutant attached to the tube surface. A total of 5 μM C16-HSL or an equivalent amount of C16-HSL associated with MVs was added. The same amount of MVs derived from the wild-type or the *pdnI* mutant was added. (**d**) MV addition to a Pd1222 AHL reporter strain. A total of 5 μM C16-HSL or an equivalent amount of C16-HSL associated with MVs was added to a culture of *P. denitrificans* Pd1222Δ*pdnI/*pPLlas. Gfp expression in this strain is dependent on C16-HSL. *n*=3; mean±s.d. Unpaired *t*-test with Welch's correction. ns, not significant; ***P<0.001. (**e**) Quantification of C16-HSL and their localization. C16-HSL was quantified from each fractions with or without ethyl acetate extractions before measurement with UHPLC-qToF-MS. *n*=3; mean±s.e. (**f**) MV induction by MMC. MMC was added at an OD_600_ of 0.5, following incubation for 5 h. MV production was measured by staining with the membrane-specific dye FM4–64. *n*=3; mean±s.d. Significant differences with the control were determined by two-way ANOVA with Dunnett's multiple comparisons post test. ns, not significant; ****P<0.0001. (**g**) C16-HSL concentration in the supernatant. MVs were induced by adding MMC as mentioned before. AHL concentration in the supernatant was measured using *C. violaceum* VIR24/pPROBE-vioA. Relative values are shown. *n*=3; mean±s.d. Significant differences were determined by two-way ANOVA with Dunnett's multiple comparisons post test. ns, not significant; ****P<0.0001.

**Figure 2:**
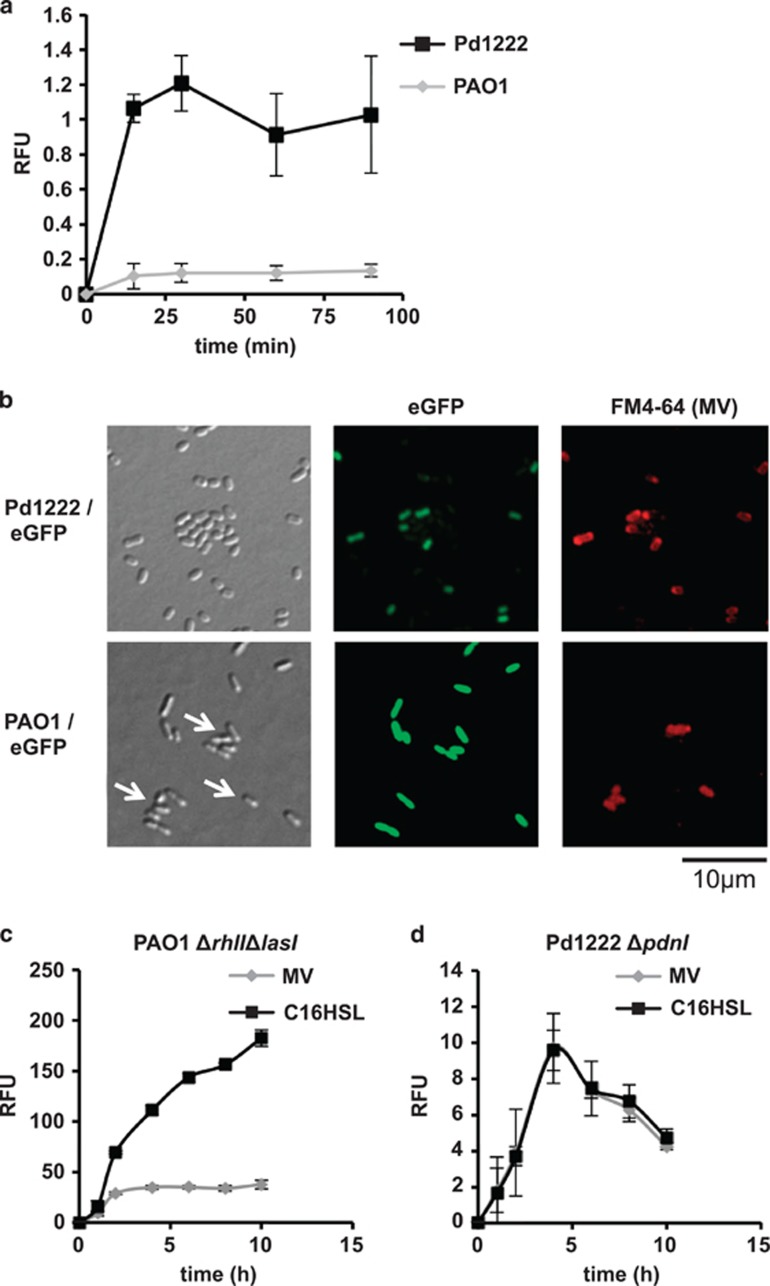
Pd1222 derived MVs traffic C16-HSL signaling. (**a**) Attachment of Pd1222 MVs to *P. denitrificans* Pd1222 or *P. aeruginosa* PAO1. Cells of *P. denitrificans* Pd1222 or *P. aeruginosa* PAO1 were mixed with FM4–64 labeled MVs and fusion of MVs to bacterial cells was quantified by measuring red fluorescence, that is, cells that had fused with the labelled MVs. *n*=3; mean±s.d. (**b**) MVs show higher affinity to *P. denitrificans* Pd1222 than *P. aeruginosa* PAO1 cells in a mixed culture. Pd1222 and PAO1 cells were mixed 1:1 and incubated in PBS for 3 h in the presence of FM4–64-labeled MVs. Pd1222 and PAO1 cells are marked with eGFP in the upper and lower panel, respectively. Upon fusion of labeled MVs with a bacterial cell, it becomes red fluorescent. In the upper panel the red fluorescence (that is the MV target cell) co-localizes with the green fluorescence of the marked Pd1222 cells, indicating that these cells are the preferred targets of the MVs. By contrast, red fluorescence does not co-localize with the green fluorescence of the labeled PAO1 cells (lower panel), indicating that the MVs have a very low affinity for these cells. However, in the lower panel, the red fluorescence co-localizes with the unmarked Pd1222 cells as indicated by the white arrows. (**c** and **d**) MVs traffic C16-HSL signals. 5 μM C16-HSL or an equivalent amount of C16-HSL associated with MVs were added to *P. aeruginosa* PAO1Δ*lasI*Δ*rhlI/*pPROBE-vioA-cviR (**c**) or *P. denitrificans* Pd1222Δ*pdnI/*pPLlas (**d**). While free C16-HSL induces both biosensors, MV-associated AHLs only induce the Pd1222 biosensor, as the MVs show little affinity for PAO1 cells. *n*=3; mean±s.d.
