# Supplementary material for: Membrane vesicle-mediated bacterial communication
Source: ISME J. 2017 Mar 10;11(6):1504–9. doi: 10.1038/ismej.2017.13 (PMC5437348; doi:10.1038/ismej.2017.13)
Supplement: Supplementary Material [file ismej201713x1.pdf]

Membrane vesicle-mediated bacterial communication

Masanori Toyofuku[1,2]*, Kana Morinaga[1], Yohei Hashimoto[3], Jenny Uhl[4], Hiroko Shimamura[1], Hideki Inaba[5], Philippe Schmitt-Kopplin[4,6], Leo Eberl[2]*, Nobuhiko Nomura[1]*.

1. Department of Life and Environmental Sciences, University of Tsukuba, Tsukuba, Ibaraki, Japan
2. Department of Plant and Microbial Biology, University of Zurich, Zürich, Switzerland.
3. Technology Research Center, Sumitomo Heavy Industries, Ltd., Yokosuka, Kanagawa, Japan
4. Helmholtz Zentrum München-German Research Center for Environmental Health, Research Unit Analytical BioGeoChemistry, Neuherberg, Germany.
5. Development and Analysis Center, Sumitomo Heavy Industries Environment Co., Ltd., Yokosuka, Kanagawa, Japan
6. Technische Universität München, Chair of Analytical Food Chemistry, Freising, Germany.

Supplementary information:

・Material and Methods
・Supplementary Figures S1-S10
・Supplementary Tables S1 and S2
・References

Materials and methods

Growth conditions

Paracoccus species were grown routinely in TSB at 37°C. *Escherichia coli*, *Pseudomonas aeruginosa* were grown in LB or TSB at 37°C. *Chromobacterium violaceum* was grown in LB at 30℃. Kanamycin was used at 50μg/ml for *E. coli, P. denitrificans* and *C. violaceum*, 200μg/ml for *P. aeruginosa*. MVs were collected from *P. denitrificans* Pd1222 cultures inoculated at an initial OD600 of 0.01 and incubated for 24h. For MV induction by MMC in *P. denitrificans* Pd1222, strains were inoculated in TSB to an initial OD600 of 0.1. When the growth reached OD600 of 0.5, MMC was added and further incubated for 5h. For the aggregation assay, strains were inoculated in TSB to an initial OD600 of 0.01 and were incubated at 30°C, 150rpm for 16h.

Bacterial strains and plasmids

Strains used in this study are listed in Supplementary Table S1. Primers used in this study are listed in Supplementary Table S2. *P. denitrificans* Pd1222 gene deletion mutants were constructed as follows. To generate a Δ*pdnI* mutant, flanking regions of the *pdnI* gene was amplified with luxIF1 / luxIR1 and luxIF2 / luxIR2 primer sets. The PCR products were mixed at equimolar proportions and used for overlap extension PCR. LuxIF0 / luxIR0 primers was used to amplify the final PCR product. The PCR product was cloned into the multicloning site of pK18mobsacB (Schäfer et al 1994), and delivered into *P. denitrificans* Pd1222 by conjugation using *E. coli* S17-1 (Simon et al 1986). The Δ*recA* strain of *P. denitrificans* Pd1222 was generated in a similar way, except recAF1 / recAR1, recAF2 / recAR2 and recAF1 / recAR2 primer pairs were used to amplify the flanking regions of *recA*. A e*GFP* expression plasmid for Pd1222, pBBRMCS-Pdn-GFP was constructed by cloning eGFP derived from pEGFP to pBBRMCS2 with Pdn_gfpF / Pdn_gfpR primers. Pdn_gfpF encodes a SD sequence taken from GAPDH (Pden_4465) before the eGFP start codon.

The 16s rRNA gene sequence of activated sludge isolates was amplified using the 27F and 1494R primers (Lane 1991). The nucleotide sequence data are available in the DDBJ / EMBL / GenBank databases under the accession numbers AB698543 to AB698558A. *gfp*-based AHL reporter pPROBE-vioA was constructed by cloning the *vioA* promoter region of *C. violaceum* ATCC12472 into pPROBE-NT. PvioAF /

PvioAR primer pair was used to amplify the *vioA* promoter region. pPROBE-vioA-cviR was constructed by cloning *cviR* from *C. violaceum* ATCC12472 into pUCP24 with cviRF1 / cviRR1 primers generating pUCP24-cviR. *CviR* was then sub-cloned with the *lac* promoter from pUCP24-cviR to pPROBE-vioA using lacF2-3 / cviRR2 primers.

The *P. denitrificans* AHL reporter was produced by delivering pPLlas to *P. denitrificans* Δ*pdnI* by conjugation (Lumjiaktase et al 2010). Activated sludge was collected from industrial wastewater plants (Sumitomo heavy industry).

MV isolation and quantification

MV was isolated and quantified as previously described (Turnbull et al 2016). Filtered supernatant was ultracentrifuged for MV isolation. As shown in Fig. 1e, this MV fraction hardly contained non-MV associated C16-HSL, and therefore was used for the MV assays. Density gradient ultracentrifugation with Optiprep (Alere Technologies AS, Norway) was used for further purification when necessary. A membrane specific dye FM4-64 (Life Technologies, USA) was used for MV quantification. NanoSight NS300 (Malvern Instruments Ltd, UK) was used for MV counting.

TEM

Purified MVs by density gradient ultracentrifugation was used for TEM analysis. MVs were stained with uranyl acetate and inspected by a JOEL JEM 2000EX transmission electron microscope (Hanaichi Ultrastructure Research Institute, Japan), as previously described (Toyofuku et al 2014).

C16-HSL detection and quantification

Violacein production of *C. violaceum* VIR24 was monitored to detect C16-HSL. *C. violaceum* VIR24 / pPROBE-vioA was used for semi-quantification assays. AHL was identified in *Paracoccus* sp. AS6 by using LC / TOF-MS (Genaris, Japan). AHL from samples were extracted by acidified ethyl acetate (Mashburn and Whiteley 2005).

C16-HSL was quantified using an UHPLC-qToF-MS method already described elsewhere (Buddrus-Schiemann et al 2014). Linear standard curves for quantification were measured within a concentration range from 0.25 µM until 10 µM using a synthetic C16-HSL standard (Cayman Chemical, Ann Arbor, USA). Culture samples were analyzed in triplicates without extraction or extracted with acidified ethyl acetate

(Mashburn and Whiteley 2005). Resulting C16-HSL concentrations of MVs were calculated as mean values of triplicates.

MV delivery assays

To examine the C16-HSL delivery through MVs, AHL reporter strains were inoculated in a 96 well plate at an initial OD600 of 1.0. C16-HSL or Pd1222 derived MVs were added at the beginning of the culture and the fluorescence of the *gfp* reporter was monitored. To assay MV attachment to cells, FM4-64 stained MVs was mixed with cells that was prepared in PBS at a concentration of OD600 = 1.0. FM4-64 stained MVs were washed three times in PBS before adding them to the cells. After incubation, cells were washed three times in PBS and after resuspending the cells in distilled water, FM4-64 was quantified with Varioskan flash multimode reader (ThermoFisher Scientific, USA). For microscopic observation, *P. denitrificans* and *P. aeruginosa* was mixed at a 1:1 ratio in PBS and incubated for 30min. Cells were washed three times and observed under an epiflourecent microscope on an agarose pad. LSM710 (Carl Zeiss, Oberkochen, Germany) mounted with a CCD camera was used and the Axiovision system was used for the operation.

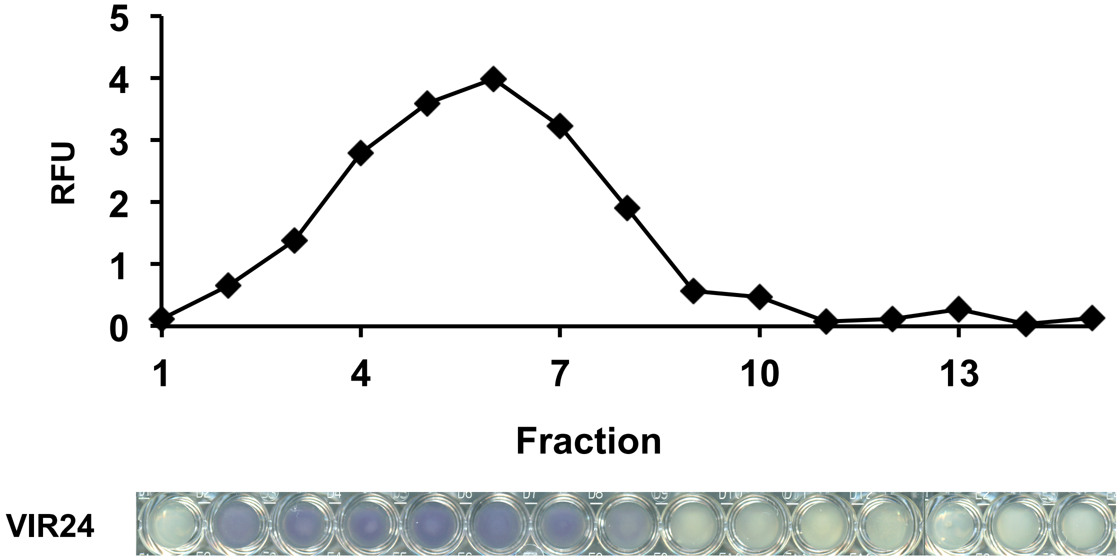

Figure S1. AHL detection of MV fractions. AHLs were detected by the aid of the AHL reporter *C. violaceum* VIR24. A sample of each of the density gradient ultracentrifugation fractions was investigated. The upper panel represents MV amount of each fraction measured by FM4-64 fluorescence and the lower panel show AHL detection with *C. violaceum* VIR24.

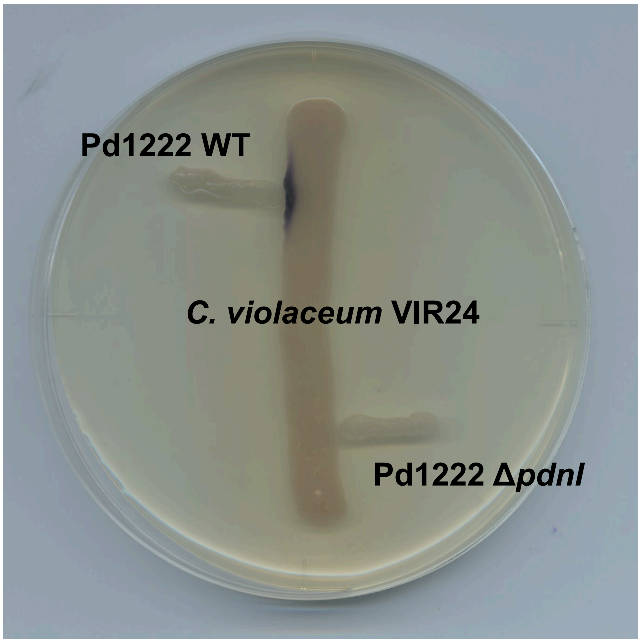

Figure S2. Inactivation of the *luxI* homolog *pdnI* abolishes AHL production of *P. denitrificans* Pd1222. The wild-type and the Δ*pdnI* mutant strain were cross-streaked against *C. violaceum* VIR24. The purple pigment produced by *C. violaceum* VIR24 is indicative of the presence of C16-HSL.

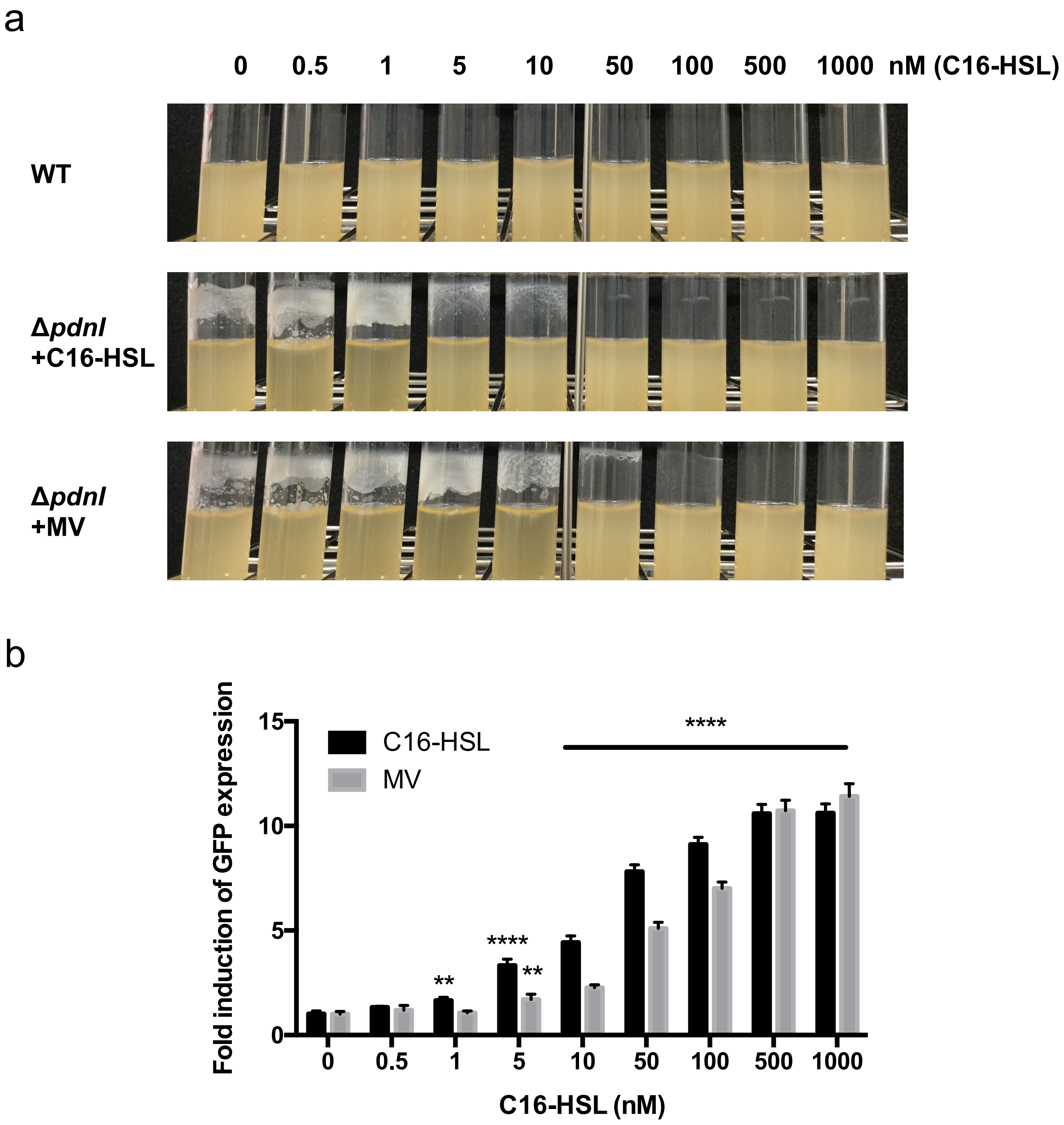

Figure S3. Response of *P. denitrificans* Pd1222 to free or MV-associated C16-HSL. (a) Inhibition of cell aggregation. C16-HSL or an equivalent amount of MV-associated C16-HSL was added to cultures of the WT or the *pdnI* mutant strain. (b) Induction of a GFP-based AHL reporter strain. C16-HSL or an equivalent amount of MV-associated C16-HSL was added to a culture of *P. denitrificans* Pd1222Δ*pdnI* / pPLlas and induction of GFP fluorescence was measured. *n*=5; mean±s.d. Significant differences were determined by two-way ANOVA with Dunnett's multiple comparisons post test. **, P < 0.01, ****, P < 0.0001.

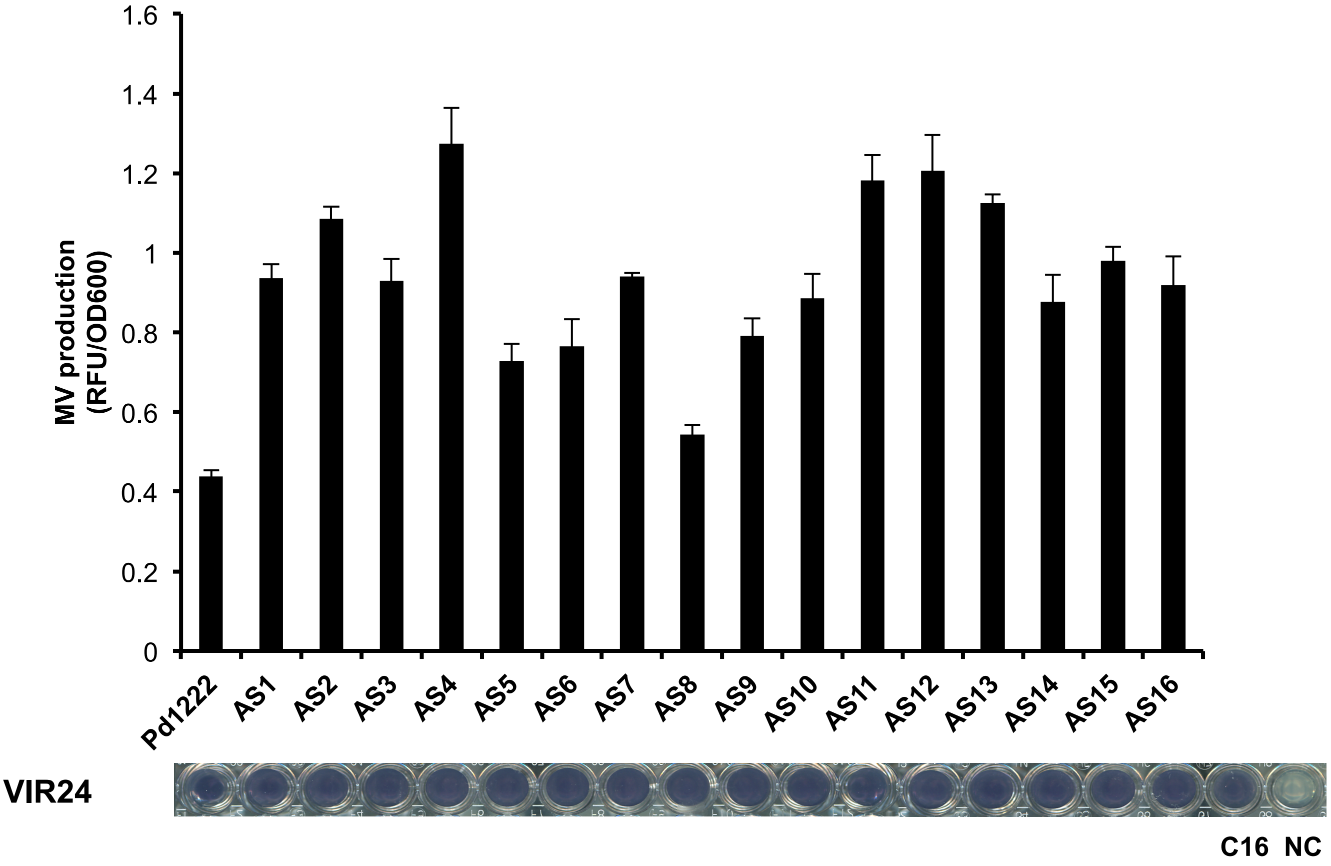

Figure S4. AHLs are associated with MVs produced by environmental isolates of *Paracoccus* sp. The upper panel shows MVs production and the lower panel show AHL detection with *C. violaceum* VIR24. *n*=3; mean±s.d.

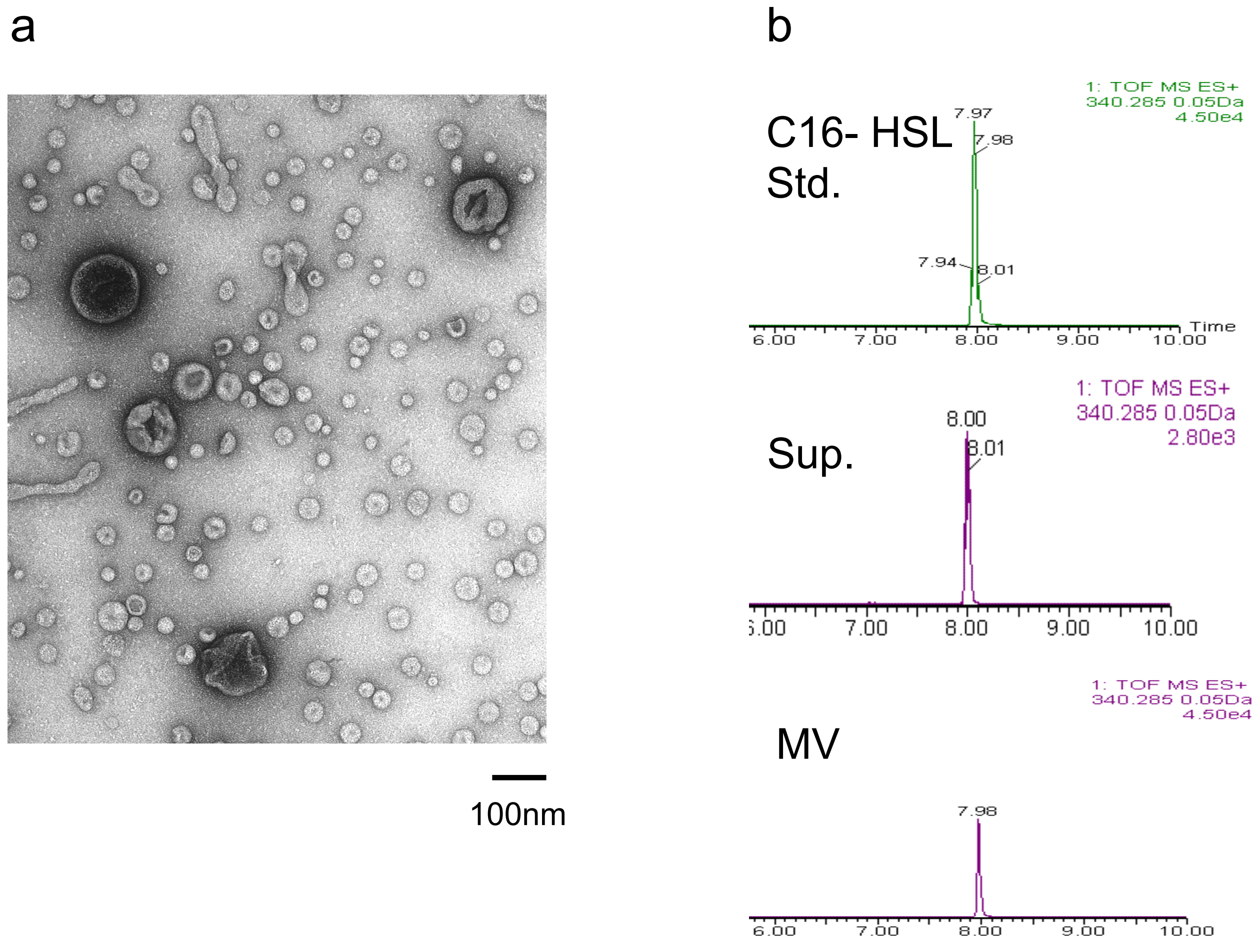

Figure S5. C16-HSL is associated with MV produced by *Paracoccus* sp. AS6. (a) MVs isolated from AS6. (b) TOF-MS analysis of C16-HSL from AS6 supernatant or MVs. Upper panel, C16-HSL standard. Middle panel, C16-HSL extracted from *Paracoccus* sp. AS6 supernatant containing MVs. Lower panel, C16-HSL extracted from MVs.

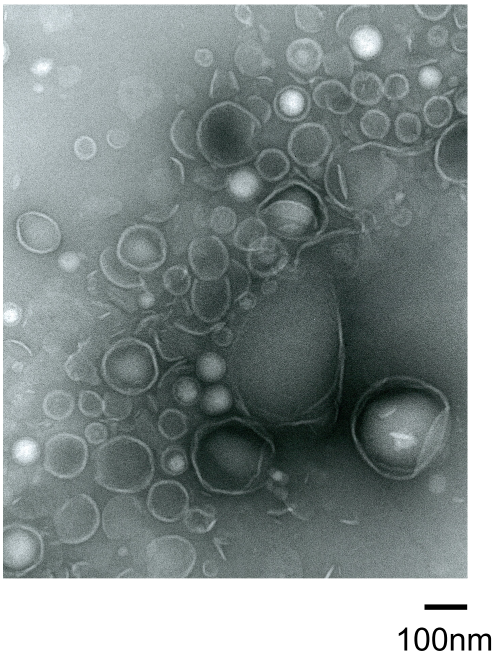

Figure S6. TEM image of MV-like structures isolated from activated sludge. MVs were purified from an activated sludge.

a

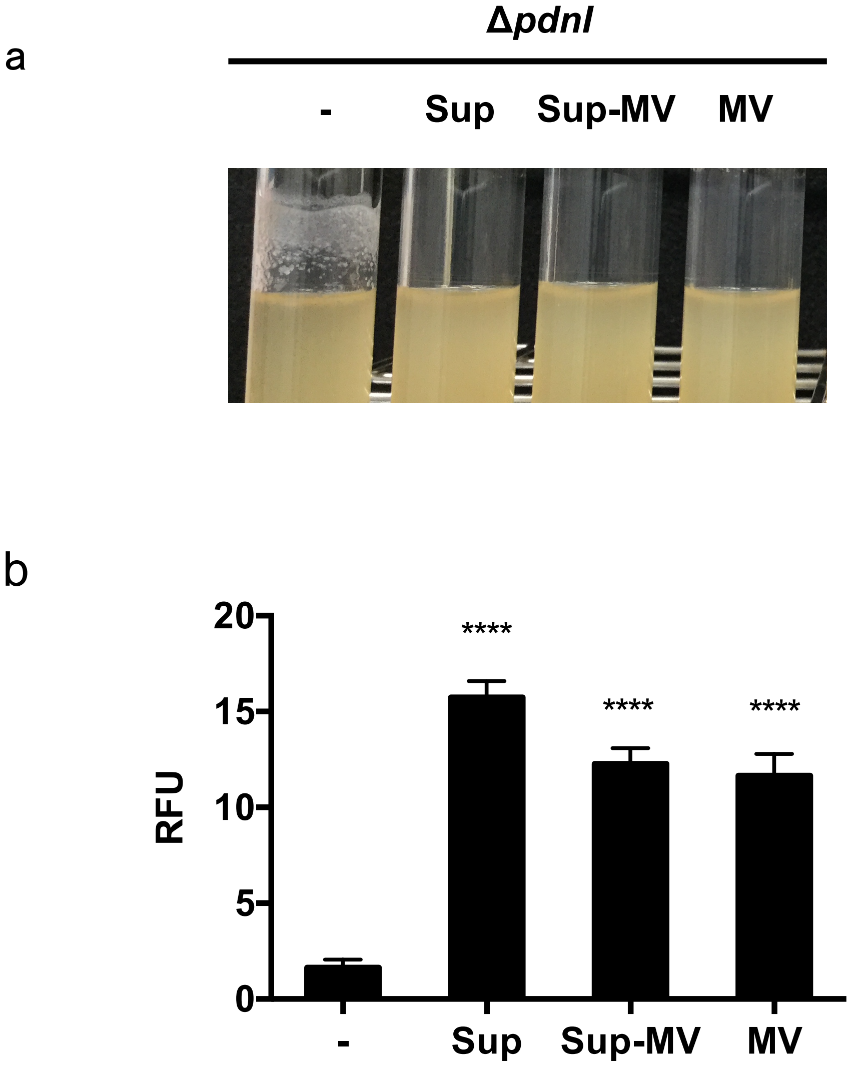

Figure S7. Response of *P. denitrificans* Pd1222 to self-produced free (Sup-MV) or MV-associated C16-HSL (MV). (a) Inhibition of cell aggregation. Free and MV-associated C16-AHL were separated by ultracentifugation of wild-type late-stationary phase culture supernatants (Sup) and were 10-fold diluted into media that was used to grow the Δ*pdnI* mutant. (b) Induction of the AHL biosensor *P. denitrificans* Pd1222 Δ*pdnI* / pPLlas by self-produced free or MV-associated C16-HSL. *n*=5; mean±s.d. Significant differences were determined by unpaired t-test with Welch's correction. ****, P < 0.0001.

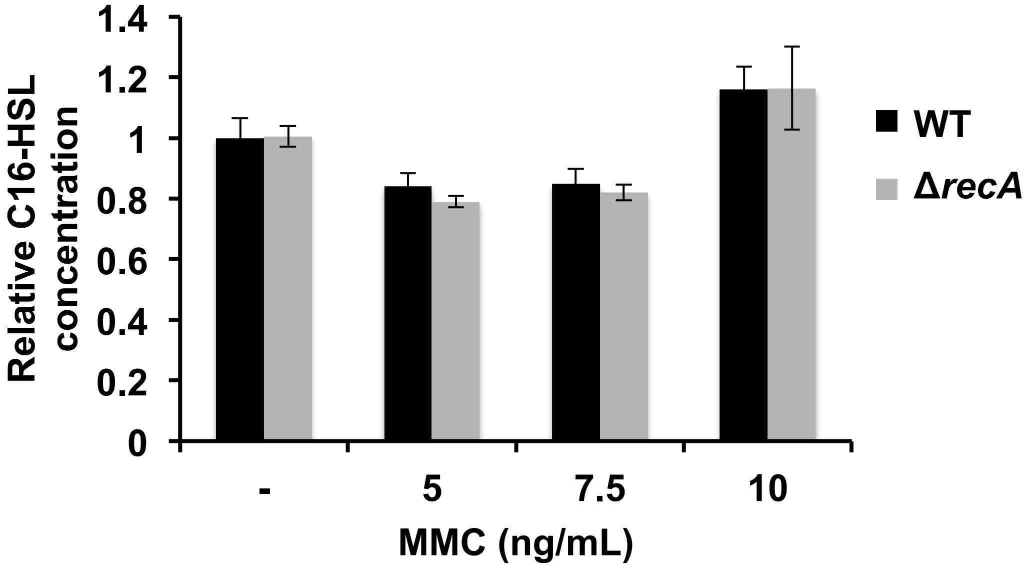

Figure S8. Relative concentration of C16-HSL in MMC-treated cultures. C16-HSL concentrations were determined by the aid of *C. violaceum* VIR24 / pPROBE-vioA, after extraction with ethyl acetate. *n*=3; mean±s.d.

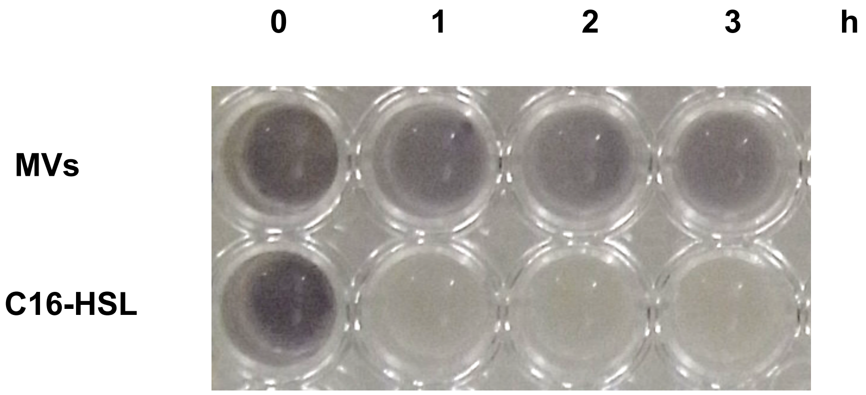

Figure S9. Recovery of C16-HSL from polypropylene tubes. C16-HSL or MVs were added to polypropylene tubes and the aqueous phase was collected every 1h. The collected samples were directly added to *C. violaceum* VIR24 lawns to detect C16-HSL.

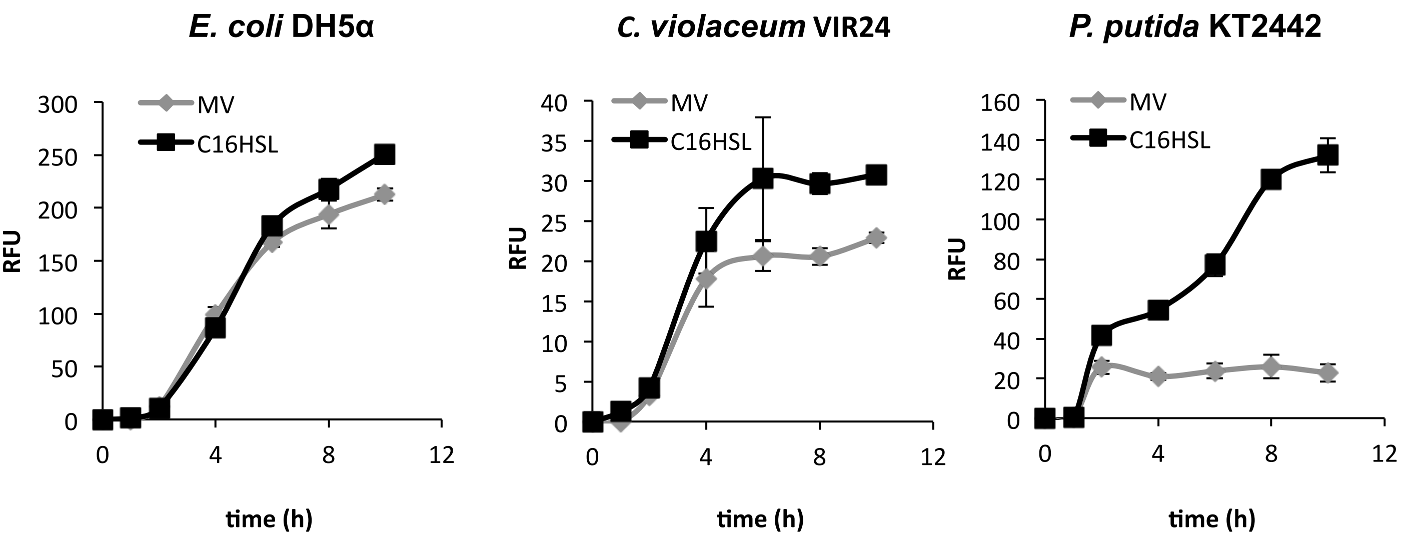

Figure S10. C16-HSL signal trafficking by MVs. 5μM C16-HSL or an equivalent amount of MV-associated C16-HSL were added to *E. coli* DH5α / pPROBE-vioA-cviR, *C. violaceum* VIR24 / pPROBE-vioA and *P. putida* KT2442 / pPROBE-vioA-cviR and GFP fluorescence was measured. *n*=3; mean±s.d.

Supplemental Table S1. List of strains and plasmids used in this study.

Strain, plasmid	Relevant characteristics	Source or reference
**Strains**		
E. coli DH5α	*E. coli* strain for transformation (F^-, *lacZ*ΔM1, *recA1*)	TaKaRa
E. coli S17-1	Mobilizer strain for conjugation	Simon et al 1986
P. denitrificans Pd1222	*P. denitrificans* type strain. Rif^r, $Spec^r$, enhanced conjugation frequencies	Devries et al 1989
P. denitrificans Δ*pdnI*	*luxI*–homolog (Pden_0787) mutant of Pd1222	This study
P. denitrificans Δ*recA*	*recA* (Pden_0597) mutant of Pd1222	This study
C. violaceum ATCC12472	*C. violaceum* type strain	Brazilian National Genome Project 2003
C. violaceum VIR24	*cviI* deletion mutant of ATCC12472	Someya et al 2009
P. aeruginosa PAO1	Wild type	Holloway
P. aeruginosa Δ*rhlI*Δ*lasI*	*rhlI* and *lasI* deletion mutant of PAO1	Toyofuku et al 2007
P. putida KT2442	Wild type, AHL negative strain, Rif^r	Herrero et al 1990
**Plasmids**		
pPROBE-NT	Promoter-probe vector; *gfp,* Km^r	Miller et al 2000
pPROBE-vioA	*vioA* promoter region fused to *gfp* in pPROBE-NT	This study
pPROBE-vioA-cviR	pPROBE-vioA carrying *cviR*	This study
pMLAC-G	*lac* promoter region fused to *egfp* in pMEXGFP	Turnbull et al 2016
pBBRMCS2	Broad host range cloning vector, Km^r	Kovach et al 1995

pBBRMCS2-Pdn-Gfp	pBBRMCS2 carrying eGFP fused to the SD sequence of GAPDH (Pden_4465)	This study
pPLlas	pUT/mini with Km^r::lasR-Plac-PlasB-gfp(ASV)-T0-T1 in the NotI site	Lumjiaktase et al 2010
pUCP24	*E. coli-Pseudomonas* shuttle vector, Gm^r	West et al 1994
pK18mobsacB	Suicide vector; *sacB* Km^r	Schäfer et al 1994
pK18-Pdn-recA	*recA* (Pden_0597) deletion cassette in pK18mobsacB	This study
pK18-Pdn-luxI	*pdnI* (Pden_0787) deletion cassette in pK18mobsacB	This study

Supplemental Table S2. Sequence of primers used in this study.

**Primers**	**Sequence 5 '→ 3' (restriction enzyme sites are underlined)**	**Source or reference**
luxIF1	TCGCTGATCGAGCATGAGCAGATCAAG	This study
luxIR1	GCCCATCAGTGCATCTTGGCTGTGGTGGTCTGCATGGTCT	This study
luxIF2	AGACCATGCAGACCACCACAGCCAAGATGCACTGATGGGC	This study
luxIR2	TTCCCACAGGGTCATGGTGTCCAG	This study
luxIF0	ACGCGTCGACCGAAACTGTTCGACGTGCT	This study
luxIR0	GGACTAGTGCCAGGATATTGACCCCATA	This study
recAF1	AACTGCAGGCTGCTTTTCGCTGTTCTTTC	This study
recAR1	CAGGATCAATCCTCGGCCATTGTTGCCCCTGCCATGCGGT	This study
recAF2	ACCGCATGGCAGGGGCAACAATGGCCGAGGATTGATCCTG	This study
recAR2	GCTCTAGACTCCAGCTTCAGCGTCTCTT	This study
PvioAF	GCTCTAGAACGCCGTTGAGGGATTGCTTGG	This study
PvioAR	GGGGTACCTCACTCCTGCTGCATGTCGAAGATG	This study
cviRF1	CGAATTCCACACAGAACCAAGAACAAGGAAGACCCG	This study
cviRR1	GGTCTAGAGCGCTGGATGTATTTCGTCGTGGAGC	This study
lacF2-3	GCTCTAGAGGCACCCCAGGCTTTACACTTTATGCTTCC	This study
cviRR2	CCCAAGCTTGCGCTGGATGTATTTCGTCGTGGAGC	This study
Pdn_gfpF	ACGGTACCCGGCGGAGGAAACCTATGGTGAGCAAGGGCGAGGA	This study
Pdn_gfpR	GCTCTAGATTACTTGTACAGCTCGTCCA	This study
27F	AGAGTTTGATCCTGGCTCAG	Lane 1991
1494R	TGACTGACTGAGGYTACCTTGTTAC	Huhe et al 2011

References

Brazilian National Genome Project C (2003). The complete genome sequence of *Chromobacterium violaceum* reveals remarkable and exploitable bacterial adaptability. *Proc Natl Acad Sci USA* **100:** 11660-11665.

Buddrus-Schiemann K, Rieger M, Mühlbauer M, Barbarossa MV, Kuttler C, Hense BA *et al* (2014). Analysis of *N*-acylhomoserine lactone dynamics in continuous cultures of *Pseudomonas putida* IsoF by use of ELISA and UHPLC/qTOF-MS-derived measurements and mathematical models. *Anal Bioanal Chem* **406:** 6373-6383.

Devries GE, Harms N, Hoogendijk J, Stouthamer AH (1989). Isolation and characterization of *Paracoccus denitrificans* mutants with increased conjugation frequencies and pleiotropic loss of a (nGATCn) DNA-modifying property. *Arch Microbiol* **152:** 52-57.

Herrero M, de Lorenzo V, Timmis KN (1990). Transposon vectors containing non-antibiotic resistance selection markers for cloning and stable chromosomal insertion of foreign genes in gram-negative bacteria. *J Bacteriol* **172:** 6557-6567.

Huhe, Nomura N, Nakajima T, Uchiyama H (2011). Assimilative and co-metabolic degradation of chloral hydrate by bacteria and their bioremediation potential. *J Biosci Bioeng* **111:** 448-453.

Kovach ME, Elzer PH, Hill DS, Robertson GT, Farris MA, Roop RM, 2nd *et al* (1995). Four new derivatives of the broad-host-range cloning vector pBBR1MCS, carrying different antibiotic-resistance cassettes. *Gene* **166:** 175-176.

Lane DJ (1991). 16S/23S rRNA Sequencing. In: Stackebrandt E, Goodfellow M (eds). *Nucleic acid techniques in bacterial systematics*. John Wiley and Sons: Chichester, U.K. pp 115-147.

Lumjiaktase P, Aguilar C, Battin T, Riedel K, Eberl L (2010). Construction of

self-transmissible green fluorescent protein-based biosensor plasmids and their use for identification of *N*-acyl homoserine-producing bacteria in lake sediments. *Appl Environ Microbiol* **76:** 6119-6127.

Mashburn LM, Whiteley M (2005). Membrane vesicles traffic signals and facilitate group activities in a prokaryote. *Nature* **437:** 422-425.

Miller WG, Leveau JH, Lindow SE (2000). Improved *gfp* and *inaZ* broad-host-range promoter-probe vectors. *Mol Plant Microbe Interact* **13:** 1243-1250.

Schäfer A, Tauch A, Jäger W, Kalinowski J, Thierbach G, Pühler A (1994). Small mobilizable multi-purpose cloning vectors derived from the *Escherichia coli* plasmids pK18 and pK19: selection of defined deletions in the chromosome of *Corynebacterium glutamicum*. *Gene* **145:** 69-73.

Simon R, O'Connell M, Labes M, Pühler A (1986). Plasmid vector for the genetic analysis and manipulation of rhizobia and other gram-negative bacteria. *Methods Enzymol* **118:** 640-659.

Someya N, Morohoshi T, Okano N, Otsu E, Usuki K, Sayama M *et al* (2009). Distribution of *N*-Acylhomoserine Lactone-Producing Fluorescent Pseudomonads in the Phyllosphere and Rhizosphere of Potato (*Solanum tuberosum* L.). *Microbes Environ* **24:** 305-314.

Toyofuku M, Nomura N, Fujii T, Takaya N, Maseda H, Sawada I *et al* (2007). Quorum sensing regulates denitrification in *Pseudomonas aeruginosa* PAO1. *J Bacteriol* **189:** 4969-4972.

Toyofuku M, Zhou S, Sawada I, Takaya N, Uchiyama H, Nomura N (2014). Membrane vesicle formation is associated with pyocin production under denitrifying conditions in *Pseudomonas aeruginosa* PAO1. *Environ Microb* **16:** 2927-2938.

Turnbull L, Toyofuku M, Hynen AL, Kurosawa M, Pessi G, Petty NK *et al* (2016).

Explosive cell lysis as a mechanism for the biogenesis of bacterial membrane vesicles and biofilms. *Nat Commun* **7:** 11220.

West SE, Schweizer HP, Dall C, Sample AK, Runyen-Janecky LJ (1994). Construction of improved *Escherichia-Pseudomonas* shuttle vectors derived from pUC18/19 and sequence of the region required for their replication in *Pseudomonas aeruginosa*. *Gene* **148:** 81-86.
