# Supplementary material for: Membrane vesicle-mediated bacterial communication
Source: ISME J. 2017 Mar 10;11(6):1504–9. doi: 10.1038/ismej.2017.13 (PMC5437348; doi:10.1038/ismej.2017.13)
Supplement: Supplementary Tables [file ismej201713x2.docx]

Supplemental Table S1. List of strains and plasmids used in this study.

|  |  |  |
| --- | --- | --- |
| Strain, plasmid | Relevant characteristics | Source or reference |
| **Strains** |  |  |
| *E. coli* DH5α | *E. coli* strain for transformation (F^-^, *lacZ*ΔM1, *recA1*) | TaKaRa |
| *E. coli* S17-1 | Mobilizer strain for conjugation | Simon et al 1986 |
| *P. denitrificans* Pd1222 | *P. denitrificans* type strain. Rif^r^, Spec^r^, enhanced conjugation frequencies | Devries et al 1989 |
| *P. denitrificans* Δ*pdnI* | *luxI*–homolog (Pden_0787) mutant of Pd1222 | This study |
| *P. denitrificans* Δ*recA* | *recA* (Pden_0597) mutant of Pd1222 | This study |
| *C. violaceum* ATCC12472 | *C. violaceum* type strain | Brazilian National Genome Project 2003 |
| *C. violaceum* VIR24 | *cviI* deletion mutant of ATCC12472 | Someya et al 2009 |
| *P. aeruginosa* PAO1 | Wild type | Holloway |
| *P. aeruginosa* Δ*rhlI*Δ*lasI* | *rhlI* and *lasI* deletion mutant of PAO1 | Toyofuku et al 2007 |
| *P. putida* KT2442 | Wild type, AHL negative strain, Rif^r^ | Herrero et al 1990 |
|  |  |  |
| **Plasmids** |  |  |
| pPROBE-NT | Promoter-probe vector; *gfp,* Km^r^ | Miller et al 2000 |
| pPROBE-vioA | *vioA* promoter region fused to *gfp* in pPROBE-NT | This study |
| pPROBE-vioA-cviR | pPROBE-vioA carrying *cviR* | This study |
| pMLAC-G | *lac* promoter region fused to *egfp* in pMEXGFP | Turnbull et al 2016 |
| pBBRMCS2 | Broad host range cloning vector, Km^r^ | Kovach et al 1995 |
| pBBRMCS2-Pdn-Gfp | pBBRMCS2 carrying eGFP fused to the SD sequence of GAPDH (Pden_4465) | This study |
| pPLlas | pUT/mini with Km^r^::lasR-Plac-PlasB-gfp(ASV)-T0-T1 in the NotI site | Lumjiaktase et al 2010 |
| pUCP24 | *E. coli-Pseudomonas* shuttle vector, Gm^r^ | West et al 1994 |
| pK18mobsacB | Suicide vector; *sacB K*m^r^ | Schäfer et al 1994 |
| pK18-Pdn-recA | *recA* ( Pden_0597) deletion cassette in pK18mobsacB | This study |
| pK18-Pdn-luxI | *pdnI* (Pden_0787) deletion cassette in pK18mobsacB | This study |
|  |  |  |

Supplemental Table S2. Sequence of primers used in this study.

|  |  |  |
| --- | --- | --- |
| **Primers** | **Sequence 5 '→ 3' (restriction enzyme sites are underlined)** | **Source or reference** |
| luxIF1 | TCGCTGATCGAGCATGAGCAGATCAAG | This study |
| luxIR1 | GCCCATCAGTGCATCTTGGCTGTGGTGGTCTGCATGGTCT | This study |
| luxIF2 | AGACCATGCAGACCACCACAGCCAAGATGCACTGATGGGC | This study |
| luxIR2 | TTCCCACAGGGTCATGGTGTCCAG | This study |
| luxIF0 | ACGCGTCGACCGAAACTGTTCGACGTGCT | This study |
| luxIR0 | GGACTAGTGCCAGGATATTGACCCCATA | This study |
| recAF1 | AACTGCAGGCTGCTTTTCGCTGTTCTTTC | This study |
| recAR1 | CAGGATCAATCCTCGGCCATTGTTGCCCCTGCCATGCGGT | This study |
| recAF2 | ACCGCATGGCAGGGGCAACAATGGCCGAGGATTGATCCTG | This study |
| recAR2 | GCTCTAGACTCCAGCTTCAGCGTCTCTT | This study |
| PvioAF | GCTCTAGAACGCCGTTGAGGGATTGCTTGG | This study |
| PvioAR | GGGGTACCTCACTCCTGCTGCATGTCGAAGATG | This study |
| cviRF1 | CGAATTCCACACAGAACCAAGAACAAGGAAGACCCG | This study |
| cviRR1 | GGTCTAGAGCGCTGGATGTATTTCGTCGTGGAGC | This study |
| lacF2-3 | GCTCTAGAGGCACCCCAGGCTTTACACTTTATGCTTCC | This study |
| cviRR2 | CCCAAGCTTGCGCTGGATGTATTTCGTCGTGGAGC | This study |
| Pdn_gfpF | ACGGTACCCGGCGGAGGAAACCTATGGTGAGCAAGGGCGAGGA | This study |
| Pdn_gfpR | GCTCTAGATTACTTGTACAGCTCGTCCA | This study |
| 27F | AGAGTTTGATCCTGGCTCAG | Lane 1991 |
| 1494R | TGACTGACTGAGGYTACCTTGTTAC | Huhe et al 2011 |
|  |  |  |
